# Retaining participants in community-based health research: a case example on standardized planning and reporting

**DOI:** 10.1186/s13063-020-04328-9

**Published:** 2020-05-11

**Authors:** Nicole L. A. Catherine, Rosemary Lever, Lenora Marcellus, Corinne Tallon, Debbie Sheehan, Harriet MacMillan, Andrea Gonzalez, Susan M. Jack, Charlotte Waddell

**Affiliations:** 1 Children’s Health Policy Centre, Faculty of Health Sciences, Simon Fraser University, Room 2431, 515 West Hastings Street, Vancouver, BC V6B 5K3 Canada; 2 School of Nursing, Faculty of Human and Social Development, University of Victoria, Victoria, BC Canada; 3 Faculty of Health Sciences, Simon Fraser University, Burnaby, BC Canada; 4 School of Nursing, Faculty of Health Sciences, McMaster University, Hamilton, ON Canada; 5 Offord Centre for Child Studies, Department of Psychiatry and Behavioral Neurosciences, Faculty of Health Sciences, McMaster University, Hamilton, ON Canada

**Keywords:** Retention, Attrition, Randomized controlled trial, Adolescents, Pregnancy, Socioeconomic disadvantage

## Abstract

**Background:**

Effective strategies for participant retention are critical in health research to ensure validity, generalizability and efficient use of resources. Yet standardized guidelines for planning and reporting on retention efforts have been lacking. As with randomized controlled trial (RCT) and systematic review (SR) protocols, *retention protocols* are an opportunity to improve transparency and rigor. An RCT being conducted in British Columbia (BC), Canada provides a case example for developing a priori retention frameworks for use in protocol planning and reporting.

**Methods:**

The BC Healthy Connections Project RCT is examining the effectiveness of a nurse home-visiting program in improving child and maternal outcomes compared with existing services. Participants (*N* = 739) were girls and young women preparing to parent for the first time and experiencing socioeconomic disadvantage. Quantitative data were collected upon trial entry during pregnancy and during five follow-up interviews until participants’ children reached age 2 years. A framework was developed to guide retention of this study population throughout the RCT. We reviewed relevant literature and mapped essential retention activities across the study planning, recruitment and maintenance phases. Interview completion rates were tracked.

**Results:**

Results from 3302 follow-up interviews (in-person/telephone) conducted over 4 years indicate high completion rates: 90% (*n* = 667) at 34 weeks gestation; and 91% (*n* = 676), 85% (*n* = 626), 80% (*n* = 594) and 83% (*n* = 613) at 2, 10, 18 and 24 months postpartum, respectively. Almost all participants (99%, *n* = 732) provided ongoing consent to access administrative health data. These results provide preliminary data on the success of the framework.

**Conclusions:**

Our retention results are encouraging given that participants were experiencing considerable socioeconomic disadvantage. Standardized retention planning and reporting may therefore be feasible for health research in general, using the framework we have developed. Use of standardized retention protocols should be encouraged in research to promote consistency across diverse studies, as now happens with RCT and SR protocols. Beyond this, successful retention approaches may help inform health policy-makers and practitioners who also need to better reach, engage and retain underserved populations.

**Trial registration:**

ClinicalTrials.gov, NCT01672060. Registered on 24 August 2012.

## Background

Longitudinal observational studies and randomized controlled trials (RCTs) provide important opportunities to explore the processes underlying human development and the influences on health over time [1]. Such studies are often complex, typically requiring substantive resources to implement as well as significant investments of time by participants. Participant attrition, or non-adherence to study protocols, can present serious threats to both the internal and external validity of results, incur substantial research costs [2–4] and influence the economic efficiency of interventions [5]. Therefore, participant retention is critical to the success of long-term health studies and requires adequate planning and resources [4, 6–8].

Attrition challenges are magnified for populations who are experiencing socioeconomic disadvantages such as young age, low income and/or limited social supports—which influence researchers’ abilities to recruit and maintain contact with participants [6]. Yet researchers often fail to identify and address barriers to participation, leading to disadvantaged groups being characterized as “hard-to-reach” [6, 9–11]. Also, in Canada and elsewhere, policy-makers and practitioners have often struggled to reach, engage and retain populations who are experiencing socioeconomic disadvantage—with the aim of reducing health disparities and improving health outcomes [6, 11, 12]. Considerable disparities persist in health and social conditions and in the provision of public services [13, 14]. A better understanding of how to reach, engage and maintain contact with these groups can inform health research, as well as the provision of health and social services, in turn contributing to reducing health inequities [10–13].

For over three decades, researchers have been recommending that participant retention should be regarded as a legitimate field of study [7, 8, 15]. Accordingly, retention research has gained more prominence [6, 16–19]. For example, a “Study Within a Trial” (SWAT) is an independent methodological study embedded within a trial to examine whether trial activities, such as specific retention efforts, are effective [20]. Yet systematic reporting of planned, theory-informed retention strategies is still lacking in long-term health research methodology [6, 20]. In comparison, transparent and systematic reporting for RCT methods has greatly improved through initiatives such as Consolidated Standards of Reporting Trials (CONSORT) and the Standard Protocol Items: Recommendations for Interventional Trials (SPIRIT) [21, 22]. CONSORT provides standardized protocols in the form of checklists, flow diagrams and relevant literature to improve the reporting of trials [23]. Similarly, standardized protocols have been developed for registering and reporting on systematic reviews, such as PRISMA (Preferred Reporting Items for Systematic Reviews and Meta-Analyses) and PROSPERO, an international prospective register of systematic reviews [24, 25]. Such guidelines are also needed to promote standardization in retention practices. One such approach is the adoption of planned retention protocols that involve systematic evaluation and reporting of strategies and that are reproducible across diverse populations and studies [20, 26, 27]. These planned retention protocols would identify strategies early in the planning process with continued efforts across the engagement/recruitment and maintenance/follow-up phases of data collection.

An RCT being conducted in British Columbia (BC), Canada provides a case example to highlight the importance of a priori retention protocol planning and standardized reporting in general, and to offer our trial as an example—to encourage the development of new collective approaches to planning and reporting, parallel to what the research community has achieved, for example, with CONSORT (for trials) and PRISMA and PROSPERO (for systematic reviews). Our specific objectives are: to describe the process of developing an evidence-informed and theory-informed participant retention framework for use in protocol planning and reporting, using the BC RCT as a case example; and to provide recommendations for planning, evaluating and reporting on participant retention across diverse populations and studies.

### BC Healthy Connections Project

The BC Healthy Connections Project (BCHCP) involves an RCT examining the effectiveness of the Nurse–Family Partnership (NFP) program compared with existing services in improving child development and mental health, as well as maternal outcomes [28, 29].

The NFP involves intensive home visitation starting prenatally and continuing until children reach age 2 years. The program focuses on the children of young, first-time mothers who are experiencing socioeconomic disadvantage [29]. An adjunctive mixed-methods process evaluation has also examined how this public health intervention was implemented and delivered across BC [30]. A second adjunctive study is examining whether the NFP can reduce biological markers of stress in a subsample (*n* = 400) of children and mothers [31].

The NFP has been studied using RCT methods for over 40 years in diverse regions in the United States in Elmira, Memphis and Denver [32]. More recently, the NFP was also studied in the Netherlands [33] and England [34].

Two retention approaches were used in these NFP trials—led by either implementers (focused on NFP retention for the intervention group) or researchers (focused on research interview completion rates for both intervention and control groups). NFP program retention efforts have typically taken the first approach, led by practitioners; in the BCHCP’s case, this involved public health nurses with NFP education. Evidence also exists on successful retention efforts led by nurses delivering the NFP in community settings in the United States [35]. The second approach has focused on research interview completion rates for the whole sample (intervention and control groups), led by researchers masked to treatment group allocation. (For long-term clinical studies, a rate of 80% or higher is considered acceptable or high retention [36]). Interview completion rates have been reported in the trial publications comparing the NFP and control groups—ranging from 60 to 83% by 24 months postpartum; and being 79% by age 15 years at long-term follow-up [32, 34, 37]. Yet specific retention strategies have not been reported for these trials.

For the BCHCP, while the NFP intervention was delivered by public health nurses, our focus here was on retention of research participants within the RCT—specifically, all intervention and control participants, across multiple data-collection time points. The research interviews were conducted by field interviewers masked to treatment allocation who reminded participants prior to each interview not to disclose their treatment group allocation. Participants were invited to complete all research interviews regardless of their level of involvement in NFP. Accordingly, this paper may have implications for all types of community-based health research involving multiple research interviews—not just clinical trials.

The BCHCP also focuses on participants experiencing socioeconomic disadvantage, who are often described as “hard-to-reach” with respect to engaging in health services and research [6, 29]. Most participants at trial entry were facing challenges such as living on low income (84% earning less than CAD$20,000 per year) or experiencing housing instability (52% had had to move three or more times in the past year). More than two-thirds (70%) were also experiencing cumulative disadvantage (defined as four or more forms of adversity) [29]. A brief summary of the data-collection schedule is described in the following. Details on the RCT, including the eligibility criteria, main outcome indicators and participant characteristics at study entry, are available elsewhere [28, 29].

### BCHCP data-collection procedures

The BCHCP RCT is being conducted over a wide geographical area, with participants living in four regional health authorities (Fraser, Interior, Island and Vancouver Coastal Health). Eligible and consenting participants (*N* = 739) were referred by public health nurses in early pregnancy, and enrolled prior to 28 weeks gestation (2013–2016). Data collection involves multiple methods and sources, including: maternal self-report surveys administered in the home or by telephone (completed in 2019); child and maternal observational and cognitive tests in the home (completed in 2019); and administrative health data available through the BC health system to inform the primary outcome indicator, childhood injuries (being completed in 2021). (Participants have provided written informed consent for us to access the BC administrative data, even if they do not complete all follow-up interviews or the intervention.)

Field interviewers located across the four health authorities conducted research interviews with individual participants, commencing in pregnancy and continuing until their child reached age 2 years. The in-person baseline interview was completed with all 739 participants. Participants were then randomly allocated to receive existing services (comparison group) or the NFP plus existing services (intervention group). In late 2019, all five post-baseline research interviews were completed for study participants: by telephone at 34 weeks gestation; and in-person or by telephone at 2, 10, 18 and 24 months postpartum. Beyond completing the RCT, retention remains important as we aim to conduct long-term follow-up on the BCHCP child cohort, across childhood and adolescence, to determine longer-term benefits that may be associated with the NFP, as was found in the United States [37, 38].

## Methods

### Literature review on participant retention protocols

As an initial step, a preliminary literature review was conducted by one of the authors (CT) with the following goals: to ascertain the quantity and quality of retention methodologies relevant to the BCHCP RCT and its study population; to explore whether theory-informed and evidence-informed retention protocols for study data collection already existed; and to inform the development of a retention planning framework [39]. The following electronic databases were searched: Medline, Cochrane Central Register of Controlled Trials CENTRAL), Cochrane Methodology Register (CMR), Cumulative Index of Nursing and Allied Health Literature (CINAHL), PsycINFO, Social Sciences Index and Science Citation Index Expanded. Searches were constructed using key words related to the terms *retention*, *attrition* and *hard-to-reach* or *vulnerable* study populations, as well as any relevant database-specific subject headings. Search results were restricted to health-related research involving humans, published in English between January 1, 1980 and May 1, 2016. Inclusion criteria limited eligible studies as follows: involved research participation only (i.e., not participation in a program or service within a study); required at least one follow-up visit past the point of enrollment; involved disadvantaged populations; included a rationale for selecting retention strategies; and considered retention prior to study completion (i.e., not simply post-hoc analysis of attrition).

This review identified 49 relevant articles describing 49 studies that were diverse in design, including regarding retention efforts and reporting. We extracted data within three main categories: study characteristics (e.g., study design and population type); follow-up procedures (e.g., number of assessment points and duration); and retention efforts (e.g., types and number of strategies). We then reported on the quality of evidence for both follow-up procedures (e.g., whether reasons for attrition were reported) and retention efforts (e.g., a priori retention protocol planning). Finally, we recorded any “lessons learned” that were shared by study authors. We then summarized the evidence in tabular form and conducted a descriptive analysis—exploring limitations in the studies as a whole, and discerning recommendations for improved participant retention practices. Our findings confirmed many limitations previously noted in the retention literature [39, 40]. Yet each of these limitations also presents an opportunity for advancing the field of research retention methodology. We then summarized these limitations and suggested recommendations for improving participant retention practices (see Table 1, adapted from [39]).

**Table 1:** Limitations and recommendations for improved participant retention practices

Limitations	Recommendations
Uncoordinated planning efforts	A priori retention protocol planning
Poor funding of retention efforts	Study budgets committed to retention
Heterogeneity in reporting	Standardized reporting
Descriptive/narrative analyses	Quantitative/comparative analyses
Post-hoc analyses of “what worked”	Ongoing evaluation of specific strategies

Adapted with permission from [39]

### Theoretical model

A retention model can provide scaffolding for identifying barriers and organizing strategies to enhance research participation [41]. To inform BCHCP planning, we selected an ecological model of research participation developed by Shumaker et al. and Marcellus, and applied by Meneses et al. and Salihu et al. [26, 41–43]. We chose this model because it was comprehensive, addressing the multiple layers of influence on research participation (participant, researcher, study and environment). This model also captures the complex interactions across these layers [41] and is generalizable across diverse studies and populations [26, 41, 43]. Furthermore, this participant-centered ecological model of retention is consistent with recent research strategies emphasizing “person-centered” or “patient-oriented” approaches in the United Kingdom, Canada and the United States [44–46].

### Adapting the ecological participant retention model

**Fig. 1:**
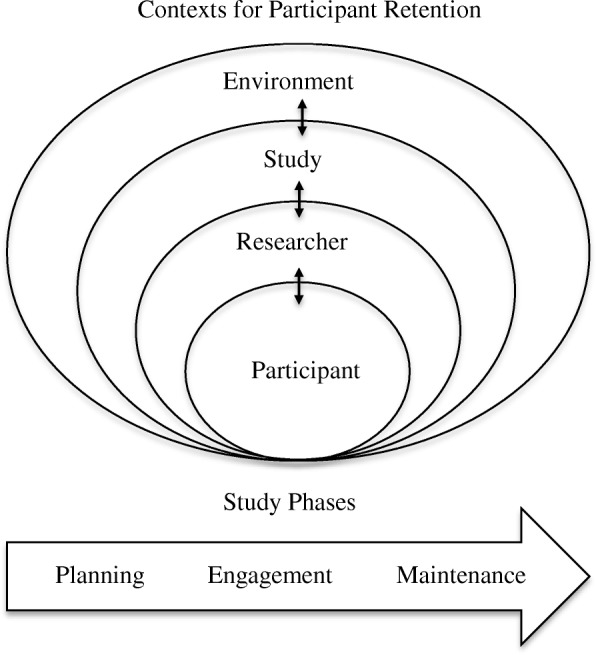
Ecological participant retention model. Adapted from Shumaker et al. and Marcellus, and applied by Meneses et al. and Salihu et al. [26, 41–43]

We adapted the ecological model for BCHCP participant retention following three study phases: planning (promote participation and prevent attrition prior to study commencement); participant recruitment and engagement (formal recruitment and early identification of non-adherence); and maintenance (long-term retention and re-engagement with those who were non-adherent). Figure 1 depicts the full model. This model then informed the development of a retention planning and reporting framework (described in the following).

### BC Healthy Connections Project retention framework

Informed by the literature review and the adapted ecological model, we then developed the BCHCP retention framework. The framework was intended to: guide the organization of strategies according to natural study phases; use “real-time” data to inform continuous evaluation; and facilitate reporting on effectiveness and costs of specific efforts. First, we identified context-specific barriers to participation (e.g., lack of up-to-date contact information for a low-income population with intermittent phone access). Then, we developed strategies based on the literature and the model to enable us to influence participants’ behavior (e.g., identifying alternative contacts, and creating protocols for re-establishing contact and participation) [27]. Finally, we organized the strategies across the planning, participant engagement and maintenance/follow-up phases. In aggregate, these strategies provide a framework for retention planning, protocol development and reporting.

## Results

### Planning phase

Planning is critical to retention and therefore is a valuable component of the research design process [41, 42]. Adequate planning also enhances participants’ ties with the researchers and the study [12, 41]. For the BCHCP adapted ecological model, planning activities began prior to study launch, as the overall protocol was being developed. This planning involved two crucial components: training and supporting research staff; and developing a robust participant-tracking system (see Table 2).

**Table 2:** Retention planning activities

1. Establish a local retention working group	
2. Identify funds within the study budget for retention	
3. Design the study to maximize participant reach, engagement and retention (see also Tables 3, 4, 5), e.g., – Develop and refine a retention protocol with theory-informed and evidence-informed strategies – Develop specific retention tools (e.g., honoraria, study fact sheets) – Develop a public study presence (e.g., name, logo, website, letterhead, business cards, toll-free participant phone line)	
4. Train and support research staff	
5. Develop a robust participant-tracking system (see also Table 3)	

#### Planning phase: training and supporting research staff

Enduring and respectful relationships between research staff and participants have been found to underlie successful retention efforts [36, 47]. Participants’ experience of being the focus of attention for research staff, in particular, is highly valued [48, 49]. This focus can provide social contact and exchange, while also giving participants a voice—which may serve as incentives for study commitment. Such incentives may be even more important for study populations such as the pregnant girls and young women participating in the BCHCP, whose circumstances may place them at risk for social isolation [50, 51].

Apart from the investigators, the BCHCP study team included senior administrative research staff, who were centrally located, and field interviewers, who traveled to participants’ homes across large geographical areas. Field interviewers typically held Bachelor’s degrees, and had relevant research and communication skills, but had no clinical training. For the BCHCP, the recruitment phase spanned 3 years and interviews were conducted over 6 years (2013–2019) with a disadvantaged study population. Therefore, we developed and provided field interviewers with 4 weeks of training covering essential topics including: research (e.g., knowledge of the study design, eligibility, measures and retention strategies); communication skills; and administration (e.g., knowledge of the participant-tracking database and processes). Field interviewers were also provided with safety training, given that data were collected in participants’ homes [3, 27, 52, 53]. (We acknowledge that this level of training may not be necessary for other studies, for example, with less disadvantaged study populations or with more experienced interviewers.)

Another common factor in long-term studies with acceptable retention rates (of 80% or more) is having a research team that not only functions well, but also is adequately supported [36]. Recognizing the need for support, we recruited full-time field interviewers to facilitate job satisfaction and reduce staff turnover, while further contributing to retention by enabling long-term participant relationships [36]. Regular support and supervision were built into overall team processes as well, through frequent team meetings and annual retention training. BCHCP field interviewers remained with the trial for an average of 3–4 years, suggesting that these efforts were successful.

#### Planning phase: developing a robust participant-tracking system

A secure, online tracking system was developed using Research Electronic Data Capture (REDCap) [54]. The BCHCP tracking system was designed to: support study operations across a large geographical area; track participants’ progress; evaluate the effectiveness of retention strategies; and track the cost of specific study processes. Accordingly, study team members (including field interviewers) used electronic data capture for all study-related information. The online system enabled sharing or masking (e.g., treatment allocation) of confidential information among study team members who were assigned varying levels of user access. We customized reports to monitor workflow and to inform retention strategies, such as reminders for upcoming interviews. Automated messages indicated time-sensitive topics requiring action (e.g., approaching missed interview deadlines). The system was designed to adapt to emerging study needs involving new information or future follow-up time points. The BCHCP retention protocol also included measures to promote research participation beyond the current study via a participant-informed consent-for-future-contact form (see Table 3).

**Table 3:** Retention planning: developing a robust participant-tracking system

Study information	Examples of data capture
Participant information	Demographics and study information for mother and child, contact information, assigned field interviewer, status changes (e.g., adoption)
Participant contacts	Mode (text, phone, email), nature (scheduling, reminder, check-in), content (date, time, contact information), frequency, time since last contact
Study status	Referral status (e.g., ineligible/declined/pending), study status (e.g., “need-to-reach”/re-engaged/withdrawn/completed)
Interviews	Type (in-person or telephone, paper or electronic), timing (within deadlines or not), nature (booked, cancelled, completed, partial, missed)
Honoraria	Gift card tracking and reconciliation
Field interviewers	Schedule and availability, participant case load, data quality checks
Communication	Among field interviewers (masked to group allocation) and onsite team
Progress reports	Monitoring recruitment and retention, generating progress reports
Randomization	Secure treatment group allocation
Retention efforts	Tracking engagement and retention materials, consent for future contact
Retention costs	Staffing hours per retention strategy (e.g., average number, frequency and type of contacts, interview mode)

### Participant engagement phase

The engagement phase is crucial in setting the stage for long-term retention. We therefore invested in promoting retention during this phase. We designed the trial so that in-person research interviews occurred early (at baseline and 2 months postpartum) to facilitate the establishment and maintenance of trusting interviewer–participant relationships—while balancing the need for data collection. Field interviewers followed a robust participant-tracking protocol throughout the 2.5 years of each family’s participation. Detailed contact information (including alternative contacts such as friend, partner or family member) was collected and verified throughout, similar to robust tracking procedures used in studies with individuals experiencing disadvantages such as homelessness or problematic substance use [27, 52]. BCHCP participants were aged 14–24 years and used cellular phone texting as their main mode of contact with the study team. Texting was therefore a central mode of contact [55]. Passive refusal (i.e., no response to different modes and frequency of contact) was anticipated and field interviewers were encouraged to persist in a respectful manner, offering flexibility around interview scheduling—which has been shown to be important with populations such as the BCHCP’s participants [27, 36, 52, 56, 57]. Table 4 depicts the participant engagement activities.

**Table 4:** Participant engagement activities

1. Referral to study team by health authorities	
– Collaborate with referral partners to obtain participant contact information, preferred modes and times of contact, consent for initial contact	
– Assign each participant to one field interviewer throughout the study, where possible	
2. Initial contact (telephone)	
– Contact participant using study cell phone with text and email functions	
– Use different modes and times of contact and obtain three alternative contacts	
– Answer questions and offer flexibility in timing and location of interview	
– Provide 24-h reminders using preferred communication mode (e.g., text messages)	
– Enquire at each encounter if there is a change in contact information	
– Accommodate interview rescheduling	
3. Baseline interview (in-person)	
– Establish rapport using effective communication techniques	
– Adopt a professional (neutral, non-judgmental) manner	
– Apply risk-mitigation training (interviewer safety)	
– Provide essential study materials outlining the research process and interview dates	
– Explain study purpose and confidentiality, answering questions and providing time to consider	
– Use visual aids to convey the type, length and timing of research interviews	
– Verify eligibility and obtain written informed consent	
– Verify contact information and alternative contacts	
– Use audio aids for survey items sensitive to reporting bias	
– Provide meaningful honoraria such as gift cards for local department stores	
– Collect information to facilitate rapport at follow-up interviews, such as preferred name	

### Maintenance phase

Long-term retention efforts involve strategies for re-engaging those who are non-adherent or non-responsive for an extended period or who request to withdraw from the study—while not being coercive [26]. To assist with these efforts, we ensured that arranging interviews was made as easy as possible for participants (e.g., offering flexibility in time and location; accommodating requests for rescheduling appointments). Some participants also required numerous contacts (five or more) to schedule an interview, with additional contacts required to yield interview completion, as other studies have found [52, 56–58]. Our participant-tracking system captured data on the number of contact attempts and the various outcomes (e.g., complete/incomplete interview), which we will summarize in future reports. We also held regular team meetings and provided annual training meetings to support field interviewers in appreciating these issues.

Field interviewers were masked to treatment allocation to ensure that the participant-tracking protocol and data-collection process were equivalent for all participants regardless of their treatment group allocation (intervention or control). Our efforts were focused on maintaining engagement in the research process, independent of the intervention (NFP). The study team also generated frequent tracking system reports to assess the overall effectiveness of retention strategies and to identify the need for new or refined approaches. For example, early in the trial we increased the frequency of “check-ins” (i.e., interviewer sent a text/email message) between each postpartum interview by 40% (from seven to ten “check-ins” over six interviews, or approximately every 2 months). We later increased the frequency of “check-ins” by an additional 20% (to 12 “check-ins” over six interviews, or approximately every 5 weeks) for individuals we defined as “need-to-reach”, that is, no interview and three contact attempts. We also implemented a specific tracking protocol for participants who we failed to reach over an extended period of time. Data on the effectiveness of these various strategies were captured to evaluate specific strategies, including costs, for future reports (see following section). Table 5 provides further details.

**Table 5:** Participant re-engagement activities

1. Follow-up interviews (in-person or telephone)	
– Emphasize the value of participant’s contribution at each interview and review the study purpose	
– Provide breaks during lengthier interviews	
2. Participant tracking and interview scheduling	
– Maintain the same field interviewer	
– Text reminders 1 week and 24 h prior to each interview	
– Attempt different modes/times/types of contact and alternative contacts (partner/friends/family)	
– Reminder of ongoing study eligibility even if previous interviews are missed	
– Offer flexibility in interview location and time and accommodate requests for rescheduling	
– Encourage participants to update team with changes in personal information	
3. Specific tracking protocols	
– Identify new approaches, vary contact type and frequency, send re-engagement letter	
4. Study team support	
– Review progress reports (completed or missed interviews, requests to withdraw)	
– Frequent field interviewer meetings and retention training	

### Evaluating the effectiveness and costs of specific strategies

The BCHCP is a long-term study which requires substantial financial investment [52, 59]. We therefore planned our retention efforts with systematic measurement and reporting to assist us in making accurate financial estimates. The BCHCP participant-tracking system has generated data on the effectiveness and costs of specific strategies for future reporting. For example, we will be able to examine the number, frequency and types of contacts that resulted in a completed interview. We can then estimate costs based on the number of staffing hours per retention strategy. Table 3 gives more details.

### BCHCP retention outcomes to date

The study team received 1177 referrals from participating public health partners and successfully “reached” and obtained an initial response from the majority (97%, or 1142) of referred participants. Of the 1177 referrals, 739 (63%) enrolled in the trial and 438 (37%) were excluded. Of the 438 excluded participants, 125 were screened and deemed ineligible, 154 were eligible and declined to participate, and 159 were passive refusals (eligibility or informed consent was not confirmed prior to passing the 28-week gestation deadline).

**Fig. 2:**
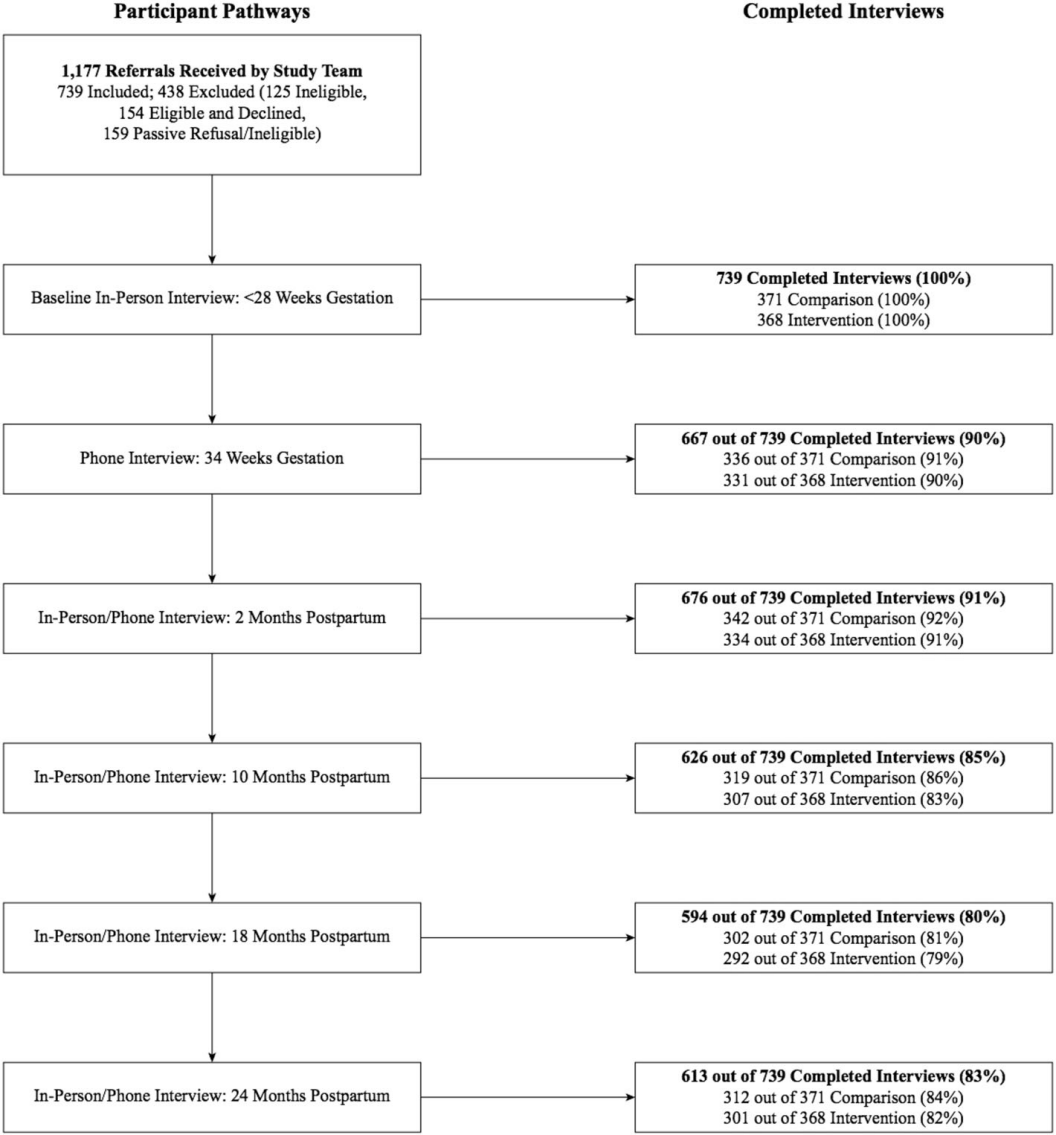
Participant interview schedule and completion rates

All 739 participants completed the baseline interview prior to 28 weeks gestation as per the trial protocol requirements. Immediately following the baseline interview, after random allocation to intervention or control groups, no participants withdrew. Results from 3302 follow-up interviews (in-person or telephone) conducted over 4 years indicate high completion rates: 90% (*n* = 667) at 34 weeks gestation prenatally; 91% (*n* = 676) at 2 months postpartum; 85% (*n* = 626) at 10 months postpartum; 80% (*n* = 594) at 18 months postpartum; and 83% (*n* = 613) at 24 months postpartum. Almost all participants (99%, *n* = 732) also provided ongoing informed consent for us to access BC administrative health data. Our results show comparable interview completion rates between participants in the control and intervention groups, despite intervention participants possibly being easier to locate due to the higher frequency of contact with the NFP nurses. These results provide preliminary data on the success of the framework and the value of retention protocol planning—including with disadvantaged populations. For further details, see Fig. 2.

Feedback from field interviewers further suggested that the following strategies were most effective: personalizing the connection, such as showing appreciation, demonstrating interest and using friendly scripts; having flexibility and working on weekends and evenings; customizing tracking protocols to change the frequency or type of contact; and being persistent (e.g., not taking non-response personally). Our field interviewers have also reported that, anecdotally, many BCHCP participants have said that they enjoy contributing to the research and having their voices heard (through the survey data)—feeling that someone is listening and is interested in their lives.

### Standardized reporting of retention protocols

The BCHCP retention framework presented here (see Tables 2, 3, 4, 5) provides an example of the content and processes that could be encouraged—and that, in aggregate, could inform the development of retention protocols for health research. Building on our retention framework, we propose that a standardized approach could be required in reporting results of RCTs or any health research involving follow-up interviews. We have developed initial recommendations that can be applied to diverse study contexts, beyond this case example, and encourage health researchers to continue to refine and adapt these steps (see Table 6). For instance, we recommend that researchers conduct a systematic review of relevant literature and utilize theoretical frameworks and participant-centered methodologies (e.g., behavior change techniques) to identify barriers/facilitators and subsequent retention strategies [20, 60, 61]. With these steps, we are encouraging health researchers to move away from the conventional post-hoc analyses of “what worked”, instead adopting a priori standardized planning and reporting for retention protocols.

**Table 6:** Recommendations for standardized planning and reporting of retention protocols

1. Developing a retention protocol	
– Conducting a systematic review of the literature	
– Using theoretical frameworks and methodologies to identify retention strategies	
– Developing retention planning, participant engagement and maintenance activities within an evaluation framework	
– Developing data-informed monitoring and evaluation of retention strategies	
2. Implementing a retention protocol	
– Evaluating and refining strategies for monitoring and assessing effectiveness	
– Conducting quantitative and qualitative comparative analyses of strategies	
– Monitoring and evaluating costs of specific efforts	
3. Publishing retention protocol and outcome data	
– Reporting in peer-reviewed journals and other public venues and sharing lessons learned	

## Discussion

Considerable literature points to the importance of participant retention in long-term health research. Yet the retention field is limited by inconsistent planning and reporting of efforts across studies [8, 41]. As a result, retention rates may be needlessly low, health research resources may be wasted and the impact of health research may be reduced. We have therefore described the development of an evidence-informed and theory-informed retention framework and offered a case example showing the feasibility of retention planning, even in highly disadvantaged populations. We have also highlighted the need for more standardized retention planning and reporting in health research in general. BCHCP retention results suggest that these efforts can yield acceptable interview completion rates [36] that are comparable to, or higher than, previous international trials comparing the NFP to (existing services) controls (ranging from 60 to 83% by 24 months postpartum) [32–34]. Data are also being generated through our study to further track retention outcomes and the effectiveness and costs associated with these efforts. We have developed high-level recommendations that health researchers can explore, refine and adapt to their study context. To truly advance the field, however, the adoption of planned retention protocols should be encouraged—for example, guided by a case example such as ours—as a necessary part of the trial funding and publication processes. Health researchers have an opportunity to collectively develop standardized checklists and guidance for retention protocol development and reporting—similar to the scientific consensus for RCTs (CONSORT) and systematic reviews (PRISMA and PROSPERO) [21–25]. A systematic and theory-informed approach can improve the validity and reliability of retention interventions, improve the quality and efficiency of studies, and provide much-needed evidence for informing retention efforts longer term.

There is an added reason for concern about retention of health research participants. As with many studies, the BCHCP study population comprises individuals who are experiencing socioeconomic disadvantage, which may limit researchers’ ability to engage and retain participants [29]. These circumstances require greater effort by researchers to understand and overcome associated barriers to participation [6, 9]. This is especially true given that poor retention has traditionally been cited as one reason for deliberately excluding disadvantaged groups from research [6]—impeding aims to reduce health inequities through research. The adoption of planned retention protocols could encourage more researchers to address this situation, thereby benefitting disadvantaged populations. Our BCHCP retention rates during pregnancy and through to 24 months postpartum are encouraging given that 70% of participants were experiencing concentrated disadvantage (four or more adversities including low income, limited education, limited social supports and housing instability) on trial entry [29].

Our purpose was to encourage the development of a priori standardized retention planning and reporting guidelines for health research. Yet we acknowledge limitations in our approach. While our review was comprehensive, it was not systematic. This work is also based on one case example. Also, we did not focus on the initial recruitment, which was conducted by our referral partners; we recommend a similar planned approach to recruitment [62]. We also did not capture reasons for individuals declining to participate in the study, which would provide valuable information on those we “need-to-reach”. Beyond this, the strategies presented in this RCT retention protocol involve relationships across multiple contexts both within and throughout all the study phases. Therefore, we did not delineate which individual strategies occur at each contextual level (i.e., environment, study, researcher or participant) for each study phase (i.e., planning, engagement/recruitment or maintenance). Also, we did not address the various methodological and ethical considerations influencing retention in health research. For example, it is possible that the relationships established between interviewer and participant may influence the therapeutic benefit of the service or program being studied. (For the BCHCP, this potential effect should be equivalent across intervention and control groups.) The intervention itself may also influence interview completion rates as well as retention within the intervention; however, no group differences in retention rates were observed in our case. Field interviewer rapport and persistence are common themes underlying successful retention efforts in long-term health research [36, 47, 52, 56, 58]. However, researchers should also monitor when persistence may be experienced as harassment, particularly with populations experiencing disadvantage.

## Conclusions

The BCHCP retention protocol is presented as a case example of a comprehensive retention framework modeled on an ecological–theoretical model of research participation. We believe that this case example provides a foundation for the development of standardized retention planning and reporting guidelines, similar to the scientific consensus approach to RCT protocols (CONSORT) and systematic reviews (PRISMA and PROSPERO), with the goal of improving the quality and efficiency of study findings. Understanding the barriers to research participation and identifying approaches to addressing these for disadvantaged populations also have the potential to inform retention efforts for programs and services. Despite Canada’s universal healthcare system, considerable disparities persist in health and social conditions and in the provision of public services [13, 14]. In Canada and elsewhere, policy-makers and practitioners have traditionally struggled to reach, engage and retain populations who are experiencing socioeconomic disadvantage—with the aim of reducing health disparities and improving health outcomes [6, 11]. We therefore hope that this case example and the arguments for improved retention planning and reporting in health research will inform a shift for researchers, policy-makers and practitioners alike—from viewing disadvantaged populations as “hard-to-reach” to viewing them as “need-to-reach”.

## Data Availability

All data generated or analyzed during this study are included in this published article. For more information, please contact the corresponding author.

## Abbreviations

BC: British Columbia
BCHCP: British Columbia Healthy Connections Project
CONSORT: Consolidated Standards of Reporting Trials Group
NFP: Nurse–Family Partnership
PRISMA: Preferred Reporting Items for Systematic Reviews and Meta-Analyses
RCT: Randomized controlled trial
REDCap: Research Electronic Data Capture
SPIRIT: Standard Protocol Items: Recommendations for Interventional Trials

## References

[1] Magnusson D, Cairns RB. Developmental science: toward a unified framework. In: Cairns RB, Elder H, Costello EJ, editors. Developmental Science. New York; Cambridge University Press. 1996:7–30.

[2] Bower P, Brueton V, Gamble C, Treweek S, Smith CT, Young B (2014). Interventions to improve recruitment and retention in clinical trials: a survey and workshop to assess current practice and future priorities. Trials.

[3] Buscemi J, Blumstein L, Kong A, Stolley MR, Schiffer L, Odoms-Young A (2015). Retaining traditionally hard to reach participants: lessons learned from three childhood obesity studies. Contemp Clin Trials.

[4] Odierna DH, Schmidt LA (2009). The effects of failing to include hard-to-reach respondents in longitudinal surveys. Am J Public Health.

[5] Timpe Z, Winokur M (2019). Integrating retention rates into economic analyses of prevention interventions. Prev Sci.

[6] Bonevski B, Randell M, Paul C, Chapman K, Twyman L, Bryant J (2014). Reaching the hard-to-reach: a systematic review of strategies for improving health and medical research with socially disadvantaged groups. BMC Med Res Methodol.

[7] Brueton V, Tierney J, Stenning S, Harding S, Meredith S, Nazareth I (2013). Strategies to improve retention in randomised trials. Cochrane Database Syst Rev.

[8] Brueton V, Tierney J, Stenning S, Rait G (2017). Identifying additional studies for a systematic review of retention strategies in randomised controlled trials: making contact with trials units and trial methodologists. Syst Rev.

[9] Shaghaghi A, Bhopal RS, Sheikh A (2011). Approaches to recruiting “hard-to-reach” populations into research: a review of the literature. Health Promot Perspect.

[10] Flanagan SM, Hancock B (2010). “Reaching the hard to reach”—lessons learned from the VCS (voluntary and community sector): a qualitative study. BMC Health Serv Res.

[11] Heaman MI, Green CG, Newburn-Cook CV, Elliott LJ, Helewa ME (2007). Social inequalities in use of prenatal care in Manitoba. J Obstet Gynaecol Can.

[12] Quinn C, Byng R, Shenton D, Smart C, Michie S, Stewart A (2018). The feasibility of following up prisoners, with mental health problems, after release: a pilot trial employing an innovative system, for engagement and retention in research, with a harder-to-engage population. Trials.

[13] Debessai Y, Costanian C, Roy M, El-Sayed M, Tamim H (2016). Inadequate prenatal care use among Canadian mothers: findings from the Maternity Experiences Survey. J Perinatol.

[14] National Collaborating Centre for Determinants of Health (2016). Common agenda for public health action on health equity.

[15] Flick SN (1988). Managing attrition in clinical research. Clin Psychol Rev.

[16] Liu Y, Pencheon E, Hunter RM, Moncrieff J, Freemantle N (2018). Recruitment and retention strategies in mental health trials—a systematic review. PLoS One.

[17] El Feky A, Gillies K, Gardner H, Fraser C, Treweek S (2018). A protocol for a systematic review of non-randomised evaluations of strategies to increase participant retention to randomised controlled trials. Syst Rev.

[18] Brunsdon D, Biesty L, Brocklehurst P, Brueton V, Devane D, Elliott J (2019). What are the most important unanswered research questions in trial retention? A James Lind Alliance Priority Setting Partnership: The PRioRiTy II (Prioritising Retention in Randomised Trials) study. Trials.

[19] Teague S, Youssef GJ, Macdonald JA, Sciberras E, Shatte A, Fuller-Tyszkiewicz M (2018). Retention strategies in longitudinal cohort studies: a systematic review and meta-analysis. BMC Med Res Methodol.

[20] Gillies K, Bower P, Elliott J, MacLennan G, Newlands RSN, Ogden M (2018). Systematic Techniques to Enhance rEtention in Randomised controlled trials: the STEER study protocol. Trials.

[21] Schulz KF, Altman DG, Moher D (2010). CONSORT 2010 statement: updated guidelines for reporting parallel group randomised trials. J Clin Epidemiol.

[22] Chan A, Tetzlaff JM, Altman DG, Laupacis A, Gøtzsche PC, Krleža-Jerić K (2013). SPIRIT 2013 statement: defining standard protocol items for clinical trials. Ann Intern Med.

[23] Moher D, Hopewell S, Schulz KF, Montori V, Gøtzsche PC, Devereaux PJ (2010). CONSORT 2010 explanation and elaboration: updated guidelines for reporting parallel group randomised trials. J Clin Epidemiol.

[24] Booth A, Clarke M, Dooley G, Ghersi D, Moher D, Petticrew M (2012). The nuts and bolts of PROSPERO: an international prospective register of systematic reviews. Syst Rev.

[25] Moher D, Liberati A, Tetzlaff J, Altman DG, Antes G, Atkins D (2009). Preferred reporting items for systematic reviews and meta-analyses: the PRISMA statement. PLoS Med.

[26] Meneses KM, Benz RL, Hassey LA, Yang ZQ, McNees MP (2013). Strategies to retain rural breast cancer survivors in longitudinal research. Appl Nurs Res.

[27] Scott CK (2004). A replicable model for achieving over 90% follow-up rates in longitudinal studies of substance abusers. Drug Alcohol Depend.

[28] Catherine N, Gonzalez A, Boyle M, Sheehan D, Jack SM, Hougham KA (2016). Improving children’s health and development in British Columbia through nurse home visiting: a randomized controlled trial protocol. BMC Health Serv Res.

[29] Catherine NLA, Lever R, Sheehan D, Zheng Y, Boyle MH, McCandless L (2019). The British Columbia Healthy Connections Project: findings on socioeconomic disadvantage in early pregnancy. BMC Public Health.

[30] Jack SM, Sheehan D, Gonzalez A, MacMillan HL, Catherine N, Waddell C (2015). British Columbia Healthy Connections Project process evaluation: a mixed methods protocol to describe the implementation and delivery of the Nurse–Family Partnership in Canada. BMC Nurs.

[31] Gonzalez A, Catherine N, Boyle M, Jack SM, Atkinson L, Kobor M (2018). Healthy Foundations Study: a randomised controlled trial to evaluate biological embedding of early-life experiences. BMJ Open.

[32] Olds DL (2008). Preventing child maltreatment and crime with prenatal and infancy support of parents: the Nurse–Family Partnership. J Scand Stud Criminol Crime Prev.

[33] Mejdoubi J, Van Den Heijkant S, Van Leerdam FJM, Heymans MW, Crijnen A, Hirasing RA (2015). The effect of VoorZorg, the Dutch Nurse–Family Partnership, on child maltreatment and development: a randomized controlled trial. PLoS One.

[34] Robling M, Bekkers MJ, Bell K, Butler CC, Cannings-John R, Channon S (2016). Effectiveness of a nurse-led intensive home-visitation programme for first-time teenage mothers (Building Blocks): a pragmatic randomised controlled trial. Lancet.

[35] Olds DL, Baca P, McClatchey M, Ingoldsby EM, Luckey DW, Knudtson MD (2015). Cluster randomized controlled trial of intervention to increase participant retention and completed home visits in the Nurse–Family Partnership. Prev Sci.

[36] Abshire M, Dinglas VD, Cajita MIA, Eakin MN, Needham DM, Himmelfarb CD (2017). Participant retention practices in longitudinal clinical research studies with high retention rates. BMC Med Res Methodol.

[37] Olds D, Henderson CR, Cole R (1998). Long-term effects of nurse home visitation on children's criminal and antisocial behavior: 15-year follow-up of a randomized controlled trial. JAMA.

[38] Olds DL, Kitzman H, Knudtson MD, Anson E, Smith JA, Cole R (2014). Effect of home visiting by nurses on maternal and child mortality: results of a 2-decade follow-up of a randomized clinical trial. JAMA Pediatr.

[39] Tallon C. Application of evidence- or theory-based retention strategies in health-related research involving “hard-to-reach” or “vulnerable” populations: a systematic review. Simon Fraser University. 2016. http://summit.sfu.ca/item/17080. Accessed 10 Mar 2020.

[40] Robinson KA, Dennison CR, Wayman DM, Pronovost PJ, Needham DM (2007). Systematic review identifies number of strategies important for retaining study participants. J Clin Epidemiol.

[41] Marcellus L (2004). Are we missing anything? Pursuing research on attrition. Can J Nurs Res.

[42] Shumaker SA, Dugan E, Bowen DJ (2000). Enhancing adherence in randomized controlled clinical trials. Control Clin Trials.

[43] Salihu HM, Wilson RE, King LM, Marty PJ, Whiteman VE (2015). Socio-ecological model as a framework for overcoming barriers and challenges in randomized control trials in minority and underserved communities. Int J MCH AIDS.

[44] Strategy for patient-oriented research. Canadian Institutes of Health Research. 2017. http://www.cihr-irsc.gc.ca/e/41204.html. Accessed 11 Mar 2020.

[45] Involve. 2017. http://www.invo.org.uk/resource-centre. Accessed 11 Mar 2020.

[46] Patient-Centered Outcomes Research Institute. 2018. https://www.pcori.org. Accessed 11 Mar 2020.

[47] Graziotti AL, Hammond J, Messinger DS, Bann CM, Miller-Loncar C, Twomey JE (2012). Maintaining participation and momentum in longitudinal research involving high-risk families. J Nurs Scholarsh.

[48] McGregor L, Parker K, LeBlanc P, King KM (2010). Using social exchange theory to guide successful study recruitment and retention. Nurse Res.

[49] Grape A, Rhee H, Wicks M, Tumiel-Berhalter L, Sloand E (2018). Recruitment and retention strategies for an urban adolescent study: lessons learned from a multi-center study of community-based asthma self-management intervention for adolescents. J Adolesc.

[50] Peel E, Parry O, Douglas M, Lawton J (2006). “It’s no skin off my nose”: why people take part in qualitative research. Qual Health Res.

[51] Katz K, El-Mohandes A, Mcneely Johnson D, Jarrett M, Rose A, Cober M (2001). Prediction of patient attrition from experimental behavioral interventions. J Community Health.

[52] Cotter RB, Burke JD, Stouthamer-Loeber M, Loeber R (2005). Contacting participants for follow-up: how much effort is required to retain participants in longitudinal studies?. Eval Program Plann.

[53] Tansey CM, Matté AL, Needham D, Herridge MS (2007). Review of retention strategies in longitudinal studies and application to follow-up of ICU survivors. Intensive Care Med.

[54] Harris PA, Taylor R, Thielke R, Payne J, Gonzales N, Conde JG (2009). Research electronic data capture (REDCap)—a metadata-driven methodology and workflow process for providing translational research informatics support. J Biomed Inform.

[55] Mitchell SG, Schwartz RP, Alvanzo AAH, Weisman MS, Kyle TL, Turrigiano EM (2015). The use of technology in participant tracking and study retention: lessons learned from a clinical trials network study. Subst Abus.

[56] Kleschinsky JH, Bosworth LB, Nelson SE, Walsh EK, Shaffer HJ (2009). Persistence pays off: follow-up methods for difficult-to-track longitudinal samples. J Stud Alcohol Drugs.

[57] Yeterian JD, Dow SJ, Kelly JF (2012). Ensuring retention in longitudinal studies: a practical evaluation of an intensive follow-up protocol and suggested adaptations. Int J Soc Res Methodol.

[58] Haley DF, Lucas J, Golin CE, Wang J, Hughes JP, Emel L (2014). Retention strategies and factors associated with missed visits among low income women at increased risk of HIV acquisition in the US. AIDS Patient Care STDs.

[59] Huynh L, Johns B, Liu SH, Vedula SS, Li T, Puhan MA (2014). Cost-effectiveness of health research study participant recruitment strategies: a systematic review. Clin Trials.

[60] Duncan EM, Bennett T, Gillies K (2020). Assessing effective interventions to improve trial retention: do they contain behaviour change techniques?. Trials.

[61] Fahim C, Hylton D, Simunovic M, Agzarian J, Finley C, Hanna WC (2019). Development of the IRIS-AR strategy: an intervention to improve rates of accrual and retention for the VTE-PRO randomized controlled trial. Trials..

[62] Huang GD, Bull J, Johnston McKee K, Mahon E, Harper B, Roberts JN (2018). Clinical trials recruitment planning: a proposed framework from the Clinical Trials Transformation Initiative. Contemp Clin Trials.

